# Response to: Modelling paternal exposure as a negative control

**DOI:** 10.1093/ije/dyaa055

**Published:** 2020-04-24

**Authors:** Mollie E Wood, Jacqueline M Cohen, Eivind Ystrom, Hedvig M E Nordeng, Sonia Hernandez-Diaz

**Affiliations:** d1 Department of Epidemiology, Harvard University T.H. Chan School of Public Health, Boston, Massachusetts, USA; d2 Department of Chronic Diseases and Ageing, Norwegian Institute of Public Health, Oslo, Norway; d3 Department of Mental Disorders, Norwegian Institute of Public Health, Oslo, Norway; d4 Department of Psychology, PROMENTA Research Center, University of Oslo, Oslo, Norway; d5 Department of Pharmacy, PharmaTox Strategic Research Initiative, Pharmacoepidemiology and Drug Safety Research Group, University of Oslo, Oslo, Norway; d6 Department of Child Health and Development, Norwegian Institute of Public Health, Oslo, Norway

We agree with Brew, *et al.*^1^ that the way we presented the paternal negative control directed acyclic graph (DAG) has the potential for confusion, and appreciate the opportunity to clarify our reasoning. Lipsitch’s original negative control DAG includes nodes for measured and unmeasured confounders L and U, with a dashed line between L and U indicating that either may cause the other, and they may share common causes.^2^ The utility of paternal exposure B as a negative control depends on the extent to which paternal exposure shares common causes with maternal exposure A.

Brew *et al*.^3^ split their confounding nodes into shared, maternal and paternal factors (1, 2 and 3), each including both measured (L) and unmeasured (U) variables. In addition, paternal exposure B may have an effect on maternal exposure, but not the converse. Incoming arrows to maternal exposure A come from L1/U1, L2/U2 and B, and incoming arrows to paternal exposure B come from L1/U1 and L3/U3. By definition then, A and B are not U comparable.

By contrast, our paper includes a single U that includes all unmeasured confounders shared by the parents.^4^ The concept of ‘sharedness’ allows us to add strength to our assumptions about U-comparability. Brew *et al*.^1^ are correct that there might be an arrow between paternal measured confounders and maternal antidepressant use, as well as with shared confounders. We separated measured confounders (L in the original Lipsitch paper) into maternal and paternal (L1 and L2) to facilitate the connection with the two steps in model adjustment. We presented a simplified DAG that omits arrows from L2 to B and U because, in order to find a biased association between paternal antidepressants and the outcome after adjustment for measured confounders, only the arrows with shared confounders were necessary. We prefer our DAG, combined with the hierarchical analytical approach used to estimate paternal negative exposure effects, as shown in Table 1 of our paper. These estimates allow readers to sequentially add control for U, L1 and L2, showing increasing attenuation of associations to demonstrate the potential for residual confounding.

However, we recognize that our choice of labels for L1 and L2, in which we equate paternal factors with being non-shared and maternal factors with being shared, invites confusion and is in need of revision. The formulation in Brew *et al.* readily lends itself to thinking carefully about multiple potentially overlooked sources of confounding, and we appreciate their bringing it to our attention. Importantly, although our papers differ in the conceptualization of splitting L and U into multiple nodes, the DAGs in both papers result in identical analytical approaches and assumptions about U-comparability.

With respect to the omitted confounders suggested by the authors, breastfeeding and indeed any postnatal factors cannot be confounders, and so are not a concern here. Maternal chronic illnesses other than depression, as well as any other unmeasured parent-specific or non-shared factor, may additionally confound the maternal effect estimates. As discussed in Lipsitch^2^ as well as our paper^4^ and the article from Brew and Gong,^3^ the paternal negative control is designed to control confounding that is a common cause of maternal exposure, paternal exposure and the outcome. We should not expect the paternal control to address other forms of confounding, and must rely on other methods, such as quantitative bias analysis.^5^

The authors additionally suggest that the results of the maternal exposure analysis do not support the need for a negative control, and further, that the results of the paternal control analysis do not suggest residual confounding. We respectfully disagree. In our study, estimates were essentially null for all antidepressants, but remained elevated for selective serotonin reuptake inhibitors for both maternal and paternal exposure models, albeit with confidence intervals that included the null. It is important to note, however, that we undertook this study in the context of multiple studies on the association between prenatal exposure to antidepressants and risk of childhood neurodevelopmental problems which have consistently observed elevated risks.^6^ Given concerns that this area of research is particularly vulnerable to confounding by indication, especially heritable risk of depression,^7^ we used data from the Norwegian Mother Father and Child Cohort Study as an example to illustrate the paternal control method for prenatal medication exposures.

The study of medication safety during pregnancy often involves confronting intractable confounding. Negative control studies are an important component of triangulating causal effects to provide bounds for sources of bias. We hope that this clarification of our paper, as well as the paper from Brew and colleagues, will prove helpful to researchers interested in applying the paternal negative control design to their own studies.

## Conflict of Interest

MW, JC, EY and HN report no conflict. SHD reports being an investigator in research grants to her institution from GSK, Eli Lilly and Takeda, and consulting for Roche and UCB.
